# The Deubiquitinase OTUB1 Is a Key Regulator of Energy Metabolism

**DOI:** 10.3390/ijms23031536

**Published:** 2022-01-28

**Authors:** Amalia Ruiz-Serrano, Christina N. Boyle, Josep M. Monné Rodríguez, Julia Günter, Agnieszka E. Jucht, Svende Pfundstein, Andreas M. Bapst, Thomas A. Lutz, Roland H. Wenger, Carsten C. Scholz

**Affiliations:** 1 Institute of Physiology, University of Zurich, 8057 Zurich, Switzerland; amalia.rserrano@gmail.com (A.R.-S.); juliaguenter@web.de (J.G.); agnieszka.jucht@uzh.ch (A.E.J.); pfundstein.s@gmail.com (S.P.); andreas.bapst@uzh.ch (A.M.B.); 2 Institute of Veterinary Physiology, University of Zurich, 8057 Zurich, Switzerland; boyle@vetphys.uzh.ch (C.N.B.); tomlutz@vetphys.uzh.ch (T.A.L.); 3 Laboratory for Animal Model Pathology (LAMP), Institute of Veterinary Pathology, University of Zurich, 8057 Zurich, Switzerland; josep.monnerodriguez@uzh.ch; 4 National Centre of Competence in Research ‘Kidney.CH’, 8057 Zurich, Switzerland

**Keywords:** deubiquitinating enzyme, energy expenditure, insulin, liver, ubiquitin system, FIH, *Hif1an*, hypoxia

## Abstract

Dysregulated energy metabolism is a major contributor to a multitude of pathologies, including obesity and diabetes. Understanding the regulation of metabolic homeostasis is of utmost importance for the identification of therapeutic targets for the treatment of metabolically driven diseases. We previously identified the deubiquitinase OTUB1 as substrate for the cellular oxygen sensor factor-inhibiting HIF (FIH) with regulatory effects on cellular energy metabolism, but the physiological relevance of OTUB1 is unclear. Here, we report that the induced global deletion of OTUB1 in adult mice (*Otub1* iKO) elevated energy expenditure, reduced age-dependent body weight gain, facilitated blood glucose clearance and lowered basal plasma insulin levels. The respiratory exchange ratio was maintained, indicating an unaltered nutrient oxidation. In addition, *Otub1* deletion in cells enhanced AKT activity, leading to a larger cell size, higher ATP levels and reduced AMPK phosphorylation. AKT is an integral part of insulin-mediated signaling and *Otub1* iKO mice presented with increased AKT phosphorylation following acute insulin administration combined with insulin hypersensitivity. We conclude that OTUB1 is an important regulator of metabolic homeostasis.

## 1. Introduction

Metabolic perturbations are associated with many common human diseases, including obesity, heart failure, diabetes and cancer [1,2]. Pharmaceutical targeting of the bioenergetic metabolism is a current focus in the development of novel treatment option for many of these diseases [2]. For the identification of possible unknown therapeutic targets, it is of key importance to improve our understanding of metabolic homeostasis as well as of the underlying signaling pathways and proteins.

The ubiquitin system conjugates ubiquitin to cellular target proteins via an elaborate functional interaction of Ub-conjugating enzymes (E1s, E2s, and E3s) [3]. Proteins of virtually all known signaling pathways are regulated by the ubiquitin system, which hence is an important contributor to cell and tissue homeostasis [3]. Poly-Ub chains can differ in the amino acid residue(s) used to interconnect Ub proteins within the chains (either via the Ub N-terminal methionine or through the seven different lysine residues), leading to distinct three-dimensional chain structures and different downstream effects [4]. K48-linked poly-ubiquitination is the most abundant Ub modification in cells, targeting modified proteins for proteasomal degradation [5]. K63-Ub poly-ubiquitination is the second most abundant Ub modification [5], serving as inducible scaffold in multiple signaling pathways [6].

Deubiquitinases (DUBs) are negative regulators of ubiquitination, counteracting it via trimming or removal of conjugated Ub chains from substrates [3,4]. DUBs are considered as potential novel therapeutic targets for various diseases [3], but the function of many of the approximately 100 known DUBs is only beginning to be unraveled. Ovarian tumor (OTU) domain-containing ubiquitin aldehyde-binding protein 1 (OTUB1) is one of the most highly expressed DUBs in cells [3,5]. OTUB1 is a unique DUB, displaying an enzymatic and a non-enzymatic function. The enzymatic activity of OTUB1 removes K48-ubiquitin chains, prolonging the half-life of the modified substrate protein [7,8]. Non-enzymatically, OTUB1 inhibits E2 enzyme activity by direct protein–protein interaction, preventing the conjugation of ubiquitin proteins to K48- and K63-linked ubiquitin chains [9,10,11,12,13,14].

We previously showed that the cellular oxygen sensor factor-inhibiting HIF (FIH) interacts with OTUB1 [15,16,17,18] and hydroxylates asparagine 22, affecting OTUB1 substrate targeting and cellular energy metabolism [18]. Furthermore, FIH and OTUB1 form a denaturation-resistant protein–protein complex, regulating OTUB1 enzymatic activity [16]. Interestingly, deletion of the FIH encoding gene *Hif1an* in mice leads to an energy metabolic phenotype, including a decreased body weight gain, decreased fat mass, increased insulin sensitivity, increased food and water intake as well as an increased O_2_ consumption, CO_2_ production and energy expenditure with a maintained respiratory exchange ratio (RER) [19]. The underlying molecular mechanisms are unclear [19].

Constitutive whole-body deletion of *Otub1* is lethal in mice during development [20,21,22], caused by perinatal asphyxiation [22]. We showed that lack of *Otub1* increases the proliferation of major parenchymal and mesenchymal cell types in the lung, reducing the available saccular air space, which likely prevents inhalation [22]. *Otub1* deletion led to an upregulation of mTOR signaling in lung tissue, which we proposed to cause the observed lethality [22]. In addition, embryonic mice lacking *Otub1* were significantly smaller and lighter compared to littermates [22], which can also be caused by increased mTOR signaling. The physiological relevance of OTUB1 is unknown in adult mice. Based on the observed effect of *Otub1* deletion on mTOR signaling during development as well as due to the reported metabolic phenotype of *Hif1an* KO mice and the shown regulation of OTUB1 by FIH, we assessed whether OTUB1 is relevant for the regulation of energy metabolism in vivo by investigating the phenotype of adult mice with induced whole-body OTUB1 deletion.

## 2. Results

### 2.1. Induced Otub1 Ablation Decreases Weight Gain and Fat Mass in Mice

**Figure 1:**
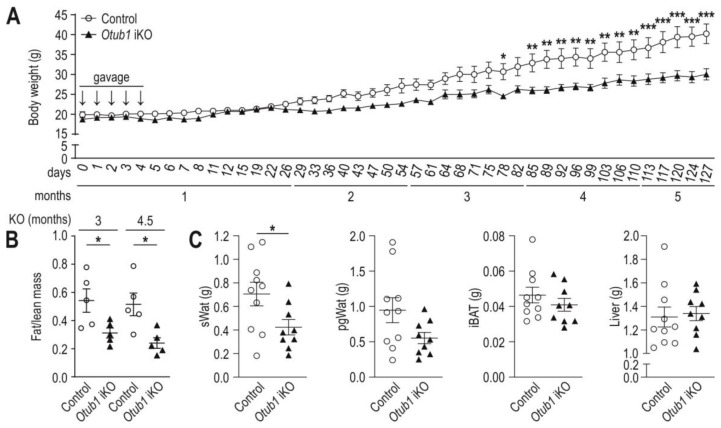
Body weight gain, fat and liver mass in mice with induced whole-body *Otub1* KO. (**A**) Body weight (BW) development following vehicle application (control) or the induction of *Otub1* KO in the whole body by tamoxifen treatment (*Otub1* iKO) in mice during months 1–5 (*n* = 5). Vehicle or tamoxifen treatment by gavage was indicated by arrows. (**B**) Fat mass to lean mass ratio analyzed by EchoMRI in the mice shown in (**A**) at the indicated time after *Otub1* KO (4.5 and 6 months of age; *n* = 5). (**C**) Weight of indicated organs (6 months after *Otub1* KO; 8.5-month-old mice; 2.5-month-old mice were gavaged with tamoxifen; control, *n* = 10; *Otub1* iKO, *n* = 9). sWAT, subcutaneous WAT; pgWAT, perigonadal WAT; iBAT, interscapular brown AT. All data are presented as the mean ± SEM. *, *p* < 0.05; **, *p* < 0.01; ***, *p* < 0.001 by two-way ANOVA with Bonferroni post-test (**A**) or two-tailed Student’s *t*-test (**B**,**C**).

Assessing possible gross phenotypes in adult mice with induced whole-body *Otub1* deletion (*Otub1* iKO), *Otub1* was ablated by tamoxifen gavage over five consecutive days and body weight (BW) progression was monitored for 122 days. The achieved KO efficiency on mRNA level was 94% in pgWAT, 77% in iBAT, 92% in liver tissue, 98% in quadriceps, 90% in lung tissue, 67% in the spleen and 96% in heart tissue (each as average from three animals) [22]. Mice without tamoxifen treatment (expressing *Otub1*; control) gained approximately 20 g within this time frame, while *Otub1* iKO mice only gained approximately 11.5 g (Figure 1A). The body composition of the same mice was assessed by EchoMRI to analyze possible causes of the reduced weight gain. The fat/lean mass ratio was decreased 3 and 4.5 months after *Otub1* KO, indicating that the amount of adipose tissue was less in *Otub1* iKO mice compared to control (Figure 1B). Analyzing various excised adipose tissues, we found that subcutaneous white adipose tissue (sWAT) weight was significantly lower in *Otub1* iKO mice and perigonadal (pgWAT) WAT showed a strong tendency towards being decreased (Figure 1C). Interscapular brown adipose tissue (iBAT) and liver weight (Figure 1C) as well as quadriceps, spleen and kidney weight (Figure S1A) were comparable between the groups, and liver tissue showed no gross histological difference (Figure S1B). In summary, these results suggest that the reduced BW gain in *Otub1* iKO mice was mainly caused by decreased WAT accumulation during aging.

### 2.2. Loss of Otub1 Leads to a Hypermetabolic State In Vivo

**Figure 2:**
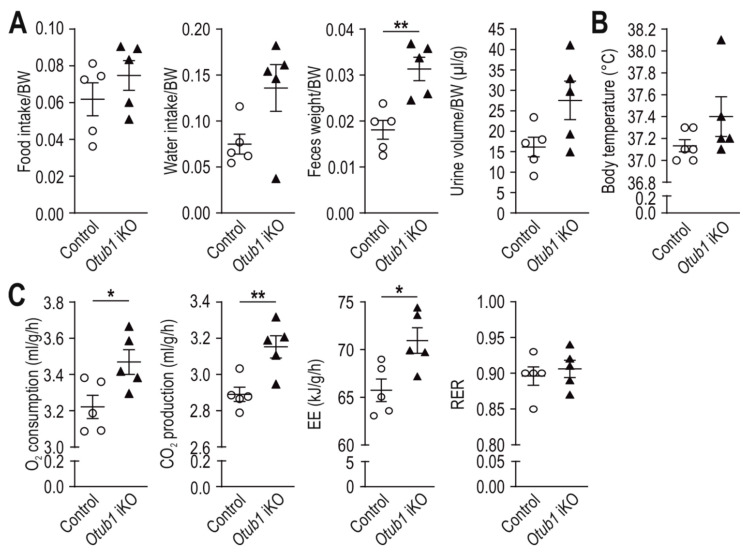
Energy metabolism in *Otub1* iKO mice. (**A**) Analysis of food and water intake, feces weight and urine volume over 72 h (4.5 months after *Otub1* KO; 6-month-old mice, *n* = 5). (**B**) Rectal body temperature (2.5 months after *Otub1* deletion; 5-month-old mice; control, *n* = 6; *Otub1* iKO, *n* = 5). (**C**) O_2_ consumption, CO_2_ production, energy expenditure (EE) and respiratory exchange ratio (RER) of 3 consecutive days during the light cycle (5 months after *Otub1* deletion; 6.5-month-old mice; *n* = 5). All data are presented as the mean ± SEM. *, *p* < 0.05; **, *p* < 0.01 by two-tailed Student’s *t*-test.

Next, we analyzed possible underlying causes of the observed BW and fat mass decrease. Food and water intake showed a tendency to be increased in *Otub1* iKO mice (Figure 2A). In agreement with a higher food and water intake, feces weight and urine volume were also increased (Figure 2A). The body temperature showed a tendency to be higher in mice lacking *Otub1* (Figure 2B). O_2_ consumption, CO_2_ production and thus energy expenditure were increased during the light cycle in *Otub1* iKO mice (Figure 2C). The RER was not altered (Figure 2C), indicating that the oxidized nutrients were the same between the groups. In the dark cycle, corresponding (albeit not significant) results were observed to the light cycle (Figure S1C). Overall, these data suggest a hypermetabolic state in *Otub1* iKO mice.

Hypermetabolism can be caused by hyperthyroidism [23], and plasma levels of thyroid-stimulating hormone (TSH) are commonly used as primary indicator for thyroid dysfunction [23]. However, plasma TSH levels were not altered between the groups (Figure S1C), indicating a normal thyroid function. Thus, we excluded hyperthyroidism as a possible cause of the observed hypermetabolic state.

### 2.3. Otub1 Deletion Increases AKT-Dependent Signaling

**Figure 3:**
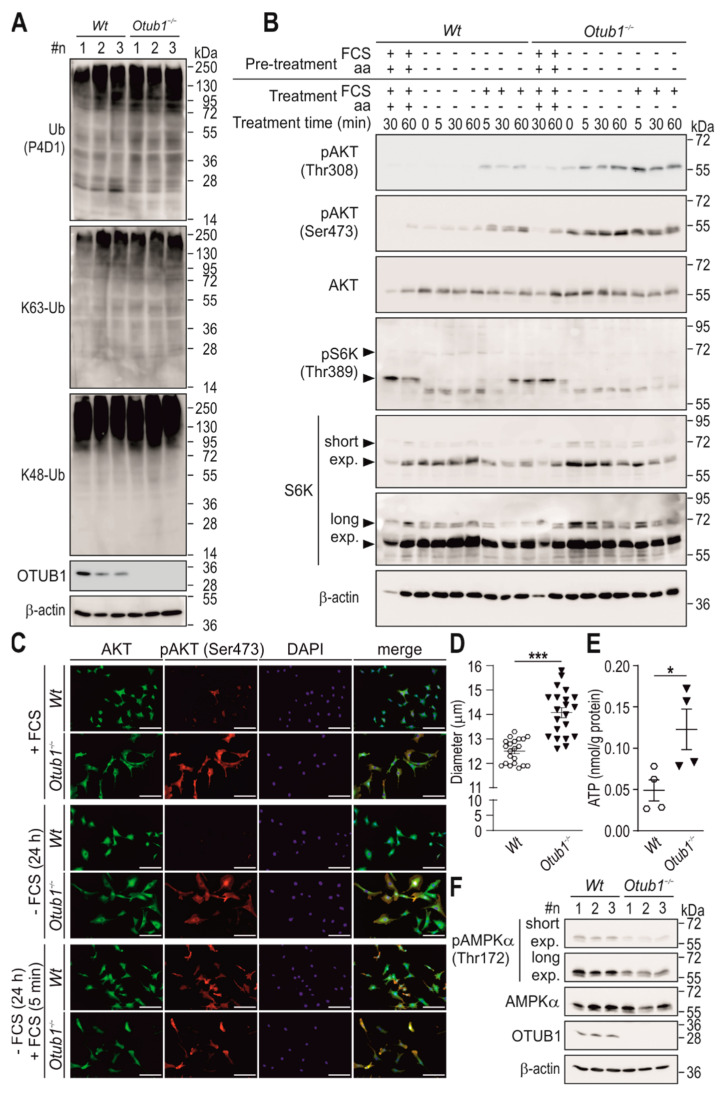
AKT/mTOR signaling in *Otub1^−/−^* MEFs. (**A**,**B**) Immunoblotting of the indicated (phospho)proteins in mouse embryonic fibroblasts (MEFs) derived from wild-type (*Wt*) and constitutive *Otub1* homozygous KO (*Otub1^−/−^*) mice (*n* = 3). K63-Ub, K63-linked ubiquitin (Ub) chains; K48-Ub, K48-linked Ub chains; FCS, fetal calf serum; aa, amino acids; pre-treatment: 4 h. (**C**) Immunofluorescence of the indicated (phospho)proteins in MEFs of the indicated genotype. Scale bar, 150 μm. (**D**) Analysis of the cell diameter of *Wt* and *Otub1^−/−^* MEFs. Each dot (*n* = 21) represents the analysis of 315 to 1197 single cells. (**E**) Intracellular ATP levels normalized to total protein content of MEFs (*n* = 4). (**F**) Immunoblotting of the indicated (phospho)proteins in *Wt* and *Otub1^−/−^* MEFs (AKT, green; pAKT (Ser473), red; DAPI, blue). exp, exposure time. Representative images are shown and data are presented as the mean ± SEM. Full-length immunoblots are shown in Figures S3–S5. *, *p* < 0.05; ***, *p* < 0.001 by two-tailed Student’s *t*-test.

To analyze possible molecular mechanisms underlying the observed changes in energy metabolism, mouse embryonic fibroblasts (MEFs) were generated from wild-type (*Wt*) mice and mice with constitutive *Otub1* deletion (*Otub1^−^*^/*−*^; Figure S2A). Despite the high cellular expression of OTUB1, total levels of Ub chains, K48-linked and K63-linked Ub chains were not altered in MEFs (Figure 3A). Therefore, we concluded that *Otub1* deletion did not affect general levels of Ub chains and we focused on the analysis of specific signaling pathways.

OTUB1 has previously been linked to the regulation of IL-15-stimulated phosphorylation of the serine/threonine kinase AKT in CD8^+^ T cells and NK cells [24] as well as to mammalian target of rapamycin (mTOR) activity through regulation of DEPTOR in vitro [25]. AKT/mTOR signaling is a major determinant of cellular energy metabolism [26,27]. Hence, we analyzed whether OTUB1 affected AKT and/or mTOR in MEFs. In *Otub1^−/−^* MEFs, both AKT Thr308 and Ser473 phosphorylation were increased under basal conditions (Figure 3B,C). Interestingly, combined depletion of FCS and amino acids (Figure 3B), or FCS depletion alone (Figure 3C), strongly reduced or abolished AKT phosphorylation in *Wt* cells. In *Otub1^−/−^* cells, AKT phosphorylation was detected in the absence of a stimulus (Figure 3B,C) and it was further enhanced in comparison to *Wt* cells after re-addition of FCS to stimulate AKT phosphorylation (Figure 3B).

To investigate mTOR activity, the phosphorylation status of the ribosomal protein S6 kinase (S6K) Thr389 was analyzed, a direct target of mTOR complex 1 (mTORC1) [26]. Under baseline and combined FCS and amino acids depletion conditions, the phosphorylation of S6K Thr389 was comparable between *Wt* and *Otub1^−/−^* cells (Figure 3B). However, following stimulation by FCS re-introduction, the phosphorylation of S6K was reduced in *Otub1*^−/−^ relative to *Wt* MEFs (Figure 3B), suggesting a decreased mTOR activity. OTUB1 has been reported to directly regulate DEPTOR protein stability [25], a negative regulator of mTORC1 and mTORC2 [28]. In *Otub1*^−/−^ MEFs, DEPTOR levels were increased (Figure S2B), inconsistent with a direct regulation of the DEPTOR protein half-life by OTUB1, but consistent with the observed decrease in mTOR activity.

To analyze the relevance of the OTUB1-dependent AKT regulation, MEF cell size and metabolic energy status were determined. We observed an increase in cell size in *Otub1^−/−^* MEFs (Figure 3C,D), in agreement with increased AKT activity [29,30]. Furthermore, ATP levels were elevated (Figure 3E) and AMPK phosphorylation was downregulated (Figure 3F), consistent with increased AKT activation [31].

### 2.4. Otub1 Deletion Increases Insulin Sensitivity

**Figure 4:**
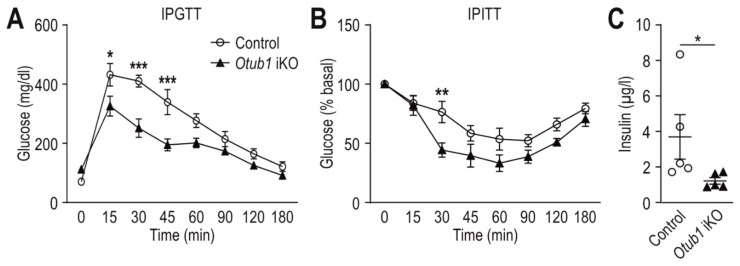
Insulin sensitivity of adult mice with induced *Otub1* KO. (**A**) Intraperitoneal (IP) glucose tolerance test (IPGTT; 2 mg glucose/g; control, *n* = 4; *Otub1* iKO, *n* = 5) and (**B**) IP insulin tolerance test (IPITT; 0.75 mU insulin/g; 4 months after *Otub1* KO; 5.5-month-old mice; *n* = 5). (**C**) Plasma insulin levels in the mice shown in (**A**) and (**B**) (5 months after *Otub1* KO; 6.5-month-old mice; *n* = 5) following 4 h of starvation during the light cycle. *, *p* < 0.05; **, *p* < 0.01; ***, *p* < 0.001 by two-way ANOVA with Bonferroni post-test (**A**,**B**) or two-tailed Mann–Whitney U test (**C**).

AKT can be activated by insulin and AKT activity plays a key role in insulin-regulated metabolism [32]. Therefore, we next assessed insulin function in *Otub1* iKO mice. In an intraperitoneal glucose tolerance test (IPGTT), *Otub1* iKO mice showed a decreased blood glucose peak and faster glucose clearance compared with control mice (Figure 4A). An intraperitoneal insulin tolerance test (IPITT) confirmed increased insulin sensitivity in *Otub1* iKO mice (Figure 4B). Furthermore, basal plasma insulin levels (after 4 h of fasting) were decreased in *Otub1* iKO mice (Figure 4C). This increased insulin sensitivity in *Otub1* iKO mice is in agreement with the increased AKT activity found in MEFs.

### 2.5. Otub1 Deletion Increases Insulin-Dependent Signaling

**Figure 5:**
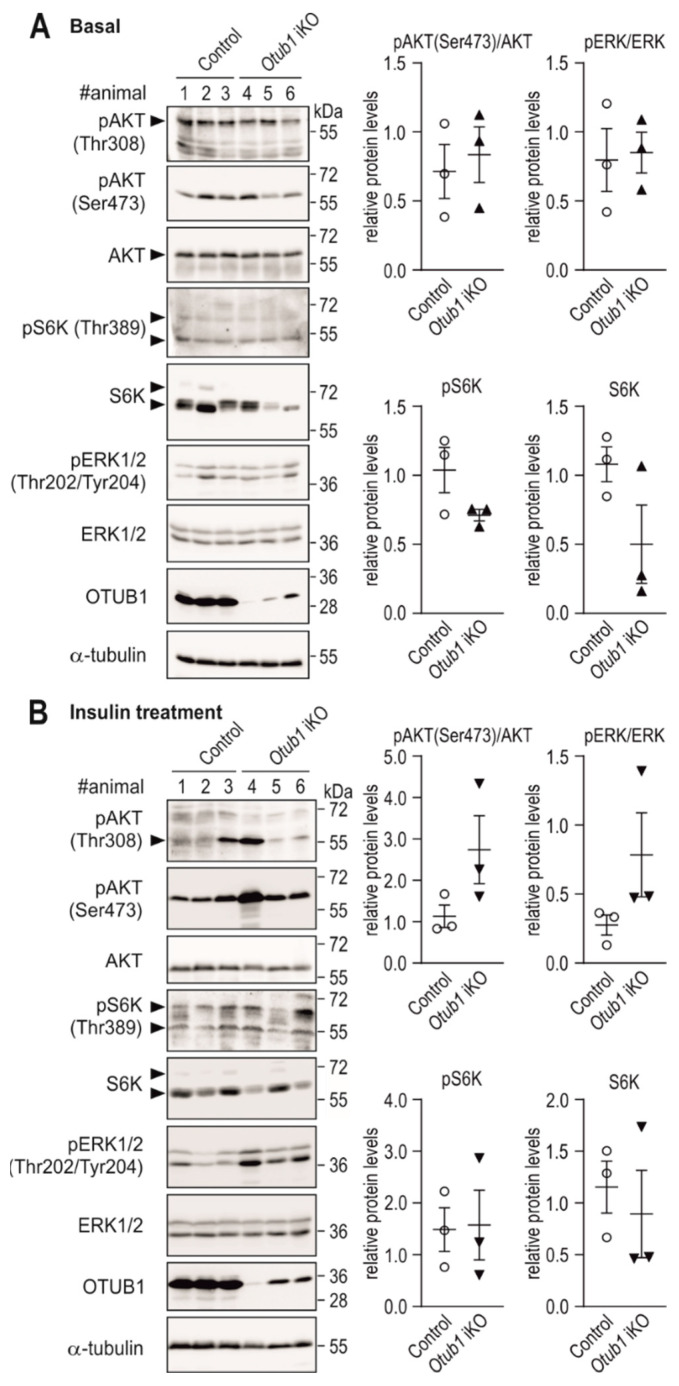
Basal and stimulated insulin receptor-dependent signaling in the liver with induced *Otub1* deletion. (**A**) Immunoblotting and quantification (normalized to loading control and the indicated proteins) of liver lysates from the mice shown in Figure 4C (*n* = 3). (**B**) Immunoblotting and quantification (normalized to loading control and the indicated proteins) of liver lysates from mice (10 days after *Otub1* KO; 2-month-old mice; *n* = 3) starved overnight (16 h) followed by intraperitoneal insulin application (0.75 mU insulin/g) 15 min prior to tissue harvest. #animal, animal number. (**A**,**B**) For pS6K, S6K, pERK1/2 and ERK1/2, both indicated immunoblot signals were quantified for the depicted quantification results. Full-length immunoblots are shown in Figures S7 and S8.

Next, we investigated insulin-dependent intracellular signaling in vivo. In the liver, no difference was observed in the phosphorylation of AKT or S6K under basal conditions (analyzed 4 h after fasting; Figure 5A and Figure S6A). There was also no difference in AKT phosphorylation in the heart under basal conditions, whereas S6K phosphorylation was increased (Figure S6B). However, the same mice had shown decreased basal insulin levels (Figure 4C). The maintained or increased AKT/mTOR signaling in the presence of decreased basal insulin levels indicated an enhanced sensitivity of AKT/mTOR signaling to insulin-dependent stimulation when *Otub1* was absent. Consequently, we next analyzed the response of AKT/mTOR signaling to acute insulin stimulation. Therefore, mice were fasted overnight followed by insulin injection. Fifteen minutes after injection, the phosphorylation of AKT on Ser473 was increased in livers of *Otub1* iKO mice (Figure 5B). The effect on AKT Thr308 was less clear (Figure 5B and Figure S6C). The phosphorylation of S6K was not altered, but total levels of S6K showed a tendency to be decreased in both basal as well as stimulated conditions (Figure 5), indicating that additional regulatory pathways may be involved. In the hearts of the same animals, phosphorylation of AKT Ser473 was at least in some animals increased in response to insulin stimulation, which could also be observed for the phosphorylation of S6K (Figure S6D).

The phosphorylation of ERK1/2, another component of the insulin signaling pathway [33], was not altered in the liver under basal conditions (Figure 5A). In response to insulin stimulation, ERK1/2 phosphorylation was increased in *Otub1* iKO livers (Figure 5B), indicating that the OTUB1-dependent regulation of insulin signaling was either not confined to AKT or occurred upstream of the bifurcation point of both signaling cascades. The phosphorylation of ERK1/2 showed a tendency to be decreased in *Otub1* iKO mice hearts following insulin stimulation, supporting a tissue-specific effect of OTUB1 on insulin-dependent signaling (Figure S6D). In summary, *Otub1* deletion led to increased insulin-dependent signaling under basal and stimulated conditions, which likely contributed to the hypermetabolic phenotype.

## 3. Discussion

Our results demonstrate that the DUB OTUB1 is essential for the homeostasis of energy metabolism and for the regulation of insulin-dependent signaling. *Otub1^−/−^* cells showed increased ATP levels and decreased AMPK phosphorylation, which is in agreement with our previous in vitro observation that OTUB1 overexpression elevates AMPK phosphorylation [18]. The here presented results suggest that OTUB1 defines a set-point for whole-body glucose metabolism in mice, with *Otub1* deletion leading to increased glucose utilization and a hypermetabolic state.

In this study, tamoxifen was used to induce the deletion of *Otub1*. Tamoxifen had previously been reported to transiently affect body weight, body composition and glucose metabolism in mice; however, such investigations were generally performed within short time frames after tamoxifen treatment (days to a few weeks) [34,35]. All of the here described in vivo results (except the analysis of insulin-mediated signaling) were obtained at least 2.5 months after tamoxifen treatment, at which time tamoxifen does not affect murine metabolism any longer (if such an effect occurs) [35,36]. Moreover, the animals in our investigations obtained tamoxifen via gavage and oral tamoxifen application does not affect glucose metabolism [34]. In addition, the observed in vivo effect of tamo-xifen-induced *Otub1* deletion on cellular signaling was reproducible in cells in vitro, which were never treated with tamoxifen (constitutive *Otub1* deletion). Thus, the here described alterations were not caused by tamoxifen treatment but by *Otub1* deletion.

*Otub1* iKO mice presented with an increased energy expenditure combined with a tendency towards a higher body temperature. This can be explained by a simultaneous increase in catabolic and anabolic metabolism and/or by mitochondrial uncoupling, which had previously been reported for *Hif1an* KO mice [19]. Future investigations will therefore need to analyze whether deletion of *Otub1* affects protein levels of enzymes that are involved in mitochondrial uncoupling, such as uncoupling proteins (UCPs). Furthermore, ATP levels were increased in MEFs, indicating an increase in catabolic cellular energy metabolism, which is in agreement with the observed increase in food intake in mice with *Otub1* deletion. Interestingly, the RER showed no difference, supporting an increased utilization of the same nutrients through the same metabolic pathways in *Otub1* iKO mice compared to control.

Lack of *Otub1* generally enhanced AKT signaling in MEF cells and in the investigated organs. AKT signaling is known to affect cellular ATP levels and AMPK phosphorylation [31], and to regulate cell size [29,30]. In adult mice, increased AKT signaling leads to decreased body weight and fat mass, increased oxygen consumption, improved glucose clearance and insulin sensitivity as well as decreased serum insulin levels [37,38]. Therefore, we conclude that OTUB1 is a negative regulator of basal and stimulated AKT signaling in cells and tissues, and that an increased AKT signaling at least partially contributes to the observed phenotypes in *Otub1* iKO mice.

Following stimulation by IL-15, OTUB1 has been reported to directly regulate AKT K63-linked ubiquitination in CD8^+^ T cells and NK cells, which is necessary for AKT membrane recruitment and the subsequent phosphorylation of AKT [24]. However, it remained unclear how exactly OTUB1 regulated IL-15-mediated AKT ubiquitination. It has previously been reported that the E2 enzymes UBC13 and/or UBCH5c are necessary for AKT ubiquitination following stimulation with insulin or epidermal growth factor (EGF) [39,40]. Both E2s can be inhibited by OTUB1 [9,12]. Hence, OTUB1 may be a negative regulator of insulin-dependent AKT ubiquitination by inhibiting UBC13 and/or UBCH5c.

Interestingly, the protein WTG1/OsOTUB1, a homologue of mouse and human OTUB1, regulates grain size and weight in rice [41,42,43]. This effect was linked to the regulation of sucrose metabolism and starch biosynthesis [41], indicating that the regulation of carbohydrate metabolism by OTUB1 was conserved during evolution.

We previously reported that the oxygen sensor FIH regulates cellular energy metabolism through hydroxylation of OTUB1 [18]. Deletion of *Hif1an* in mice led to a metabolic phenotype, but the underlying molecular mechanisms remained to be elucidated [19]. Remarkably, the reported phenotype of the *Hif1an* KO mice is virtually identical with our observations in *Otub1* iKO mice (decreased body weight gain, decreased fat mass, increased insulin sensitivity, increased food and water intake, increased O_2_ consumption, CO_2_ production and energy expenditure, a maintained RER, increased intracellular ATP levels, decreased pAMPKα levels), leading to the hypothesis that OTUB1 is a physiologically relevant target of FIH.

In summary, OTUB1 plays an essential role for the regulation of cellular and whole-body energy metabolism, at least in part through the regulation of AKT activity and insulin signaling. Our results highlight the physiological relevance of this DUB, indicate that OTUB1 is a relevant target protein of FIH in vivo and suggest that OTUB1 may be a novel therapeutic target for the treatment of type 2 diabetes, which needs to be investigated further in the future.

## 4. Materials and Methods

### 4.1. Mice

To obtain mice with induced global *Otub1* deletion, mice were obtained from the EUCOMM consortium http://www.mousephenotype.org (accessed on 4 January 2022) [44] via the EMMA repository https://www.infrafrontier.eu/ (accessed on 4 January 2022) and provided by the Institut Clinique de la Souris, ICS, Illkirch, France) as well as from The Jackson Laboratory (www.jax.org/, accessed on 4 January 2022), and crossed as previously described [22]. For our analyses, female mice (littermates) were randomly selected and gavaged with vehicle or 200 μg tamoxifen/g (Sigma-Aldrich, St. Louis, MO, USA) at 1.5 months of age (if not specified otherwise). Standard chow diet (Provimi Kliba, Gossau, Switzerland) and water were provided ad libitum, with 12 h light/dark cycles at 21 ± 1 °C under optimal hygienic conditions (OHC), unless indicated otherwise. For the assessment of insulin-dependent signaling in vivo, 0.75 U/kg insulin were intraperitoneally injected, following an overnight fasting period. Organs were harvested 15 min after injection and proteins were extracted. Investigators were blinded during analysis of in vivo data. Animal experiments were approved by the veterinary office of the canton Zurich, Switzerland (license numbers ZH035/16 [approved June 2016], ZH095/19 [approved December 2019]) and all experiments were performed in accordance with the relevant guidelines and regulations.

### 4.2. Cell Culture

Mouse embryonic fibroblasts (MEFs) were isolated from wild-type (C57BL/6N, Charles River, MA, USA) and *Otub1^−/−^* mice (obtained by crossing of heterozygous *C57BL/6N-Otub1^tm1b(EUCOMM)Hmgu/H^* mice; purchased via the EMMA repository, from the EUCOMM consortium) at day 12.5 of gestation. The Mary Lyon Centre at MRC Harwell (http://www.har.mrc.ac.uk, accessed on 4 January 2022; UK) provided Mouse Contract Services. MEFs were kept in culture until they spontaneously immortalized. MEFs were maintained in high-glucose DMEM containing 10% heat-inactivated fetal calf serum (FCS; Gibco by Life Technologies, Carlsbad, CA, USA), 1% GlutaMAX (Gibco), 200 μM 2-mercaptoethanol (Gibco), 100 μg/mL streptomycin and 100 U/mL penicillin (Sigma-Aldrich) in a humidified atmosphere at 37 °C with 5% O_2_ and 5% CO_2_. For FCS deprivation, MEFs were grown in DMEM as described above, but in the absence of FCS. For the reintroduction of FCS, the medium was exchanged with the same DMEM, but including 10% FCS. For combined FCS and amino acid deprivation, MEFs were kept in RPMI 1640 medium containing glucose but no FCS or amino acids for 4 h. To reintroduce FCS, the medium was exchanged with RPMI 1640 containing 10% FCS and the cells were harvested at the indicated time points. Cells were regularly tested for potential contamination with mycoplasma.

### 4.3. Protein Extraction

Lysis buffer for protein extraction contained 150 mM NaCl, 1 mM EDTA, 25 mM Tris-HCl pH 8.0, 1% NP-40, 1 mM Na_3_VO_4_, 1 mM PMSF and 1 mM NaF. For lysis of cells, protease inhibitor cocktail (Sigma-Aldrich) was included in the lysis buffer. For lysis of tissue, 1 μg/mL pepstatin A, 1 μg/mL aprotinin and 1 μg/mL leupeptin were added to the lysis buffer. A polytron homogenizer (VWR International, Amsterdam, The Netherlands) was used to homogenize tissue samples. For the analysis of poly-ubiquitin chains, the deubiquitinase inhibitor [45] N-ethylmaleimide (50 mM; Sigma-Aldrich) was added to the lysis buffer. The BCA assay (Thermo Fisher Scientific, Waltham, MA, USA) was used to determine protein concentrations.

### 4.4. ATP Assay

Intracellular ATP levels were measured with the CellTiter-Glo 2.0 Assay according to the manufacturer’s description (Promega, Madison, WI, USA). Cells were lysed with Passive Lysis Buffer (Promega) and diluted 1:100 in cell culture media. Samples were mixed with equal amounts of CellTiter-Glo 2.0 reagent for 2 min on an orbital shaker followed by additional 10 min of incubation at room temperature. Luminescence was determined in a microplate luminometer (Berthold Technologies, Bad Wildbach, Germany) and normalized to protein concentration determined by the Bradford method [46].

### 4.5. Immunoblot Analysis

Equal protein amounts were separated by SDS-PAGE and electro-transferred to nitrocellulose or polyvinylidene difluoride membranes. Specific proteins were detected using the following antibodies: anti-OTUB1 (Cell Signaling, Danvers, MA, USA; 3783; 1:1000), anti-p70 S6 kinase (Cell Signaling; CST9202; 1:1000), anti-phospho-p70 S6 kinase (Cell Signaling; 9234; 1:1000), anti-DEPTOR (Novus Biologicals; nbp1-49674; 1:1000), anti-AKT (pan; Cell Signaling; 2920; 1:1000), anti-phospho-Akt (Ser473; Cell Signaling; 4060; 1:1000), anti-phospho-AKT (Thr308; Cell Signaling; 2965; 1:1000), anti-p44/42 MAPK (Erk1/2; Cell Signaling, 4695), anti-phospho-p44/42 MAPK (Erk1/2; Thr202/Tyr204; Cell Signaling; 4376), anti-AMPK (Cell Signaling; 2603; 1:1000), anti-phospho-AMPK (Thr172; Cell Signaling; 2535; 1:1000), anti-poly-ubiquitin (clone P4D1; Cell Signaling; 3936; 1:1000), anti-Lys48-linked poly-ubiquitin chains (Millipore, Darmstadt, Germany; 05-1307; 1:1000), anti-Lys-63-linked poly-ubiquitin chains (Millipore; 05-1308; 1:1000), anti-β-actin (Sigma-Aldrich; A5441; 1:5000), anti-α-tubulin (Cell Signaling; 2144; 1:1000), and horseradish peroxidase (HRP)-coupled secondary antibodies (Thermo Fisher Scientific; 31,430, 31,460; 1:5000). Chemiluminescence was achieved with Supersignal West Dura (Thermo Fisher Scientific) and detected with a CCD camera (LAS-4000; GE Healthcare, Chalfont, St. Giles, UK) as described previously [47]. ImageQuant TL gel analysis software (GE Healthcare, Version 8.1) was used for quantification.

### 4.6. Immunofluorescence

Cells were fixed in 4% paraformaldehyde (Sigma-Aldrich) for 20 min at room temperature and permeabilized with 0.5% saponine (Sigma-Aldrich) in PBS. Following incubation in 5% bovine serum albumin (BSA) with 0.1% saponine for 20 min at room temperature to block unspecific protein–protein interactions, specific cellular proteins were detected using anti-AKT (pan; Cell Signaling; 2920; 1:50), anti-phospho-AKT (Ser473; Cell Signaling; 4060; 1:400) and secondary antibodies coupled with Alexa Fluor 488 (Thermo Fisher Scientific; A11001; 1:500) or Alexa Fluor 568 (Thermo Fisher Scientific; A11011; 1:500). DNA was stained with DAPI (1 μg/mL; Sigma-Aldrich) for 20 min and cells were mounted in Mowiol (Millipore) and DABCO (Sigma-Aldrich) medium. Fluorescence was recorded using an Eclipse Ts2R microscope (Nikon, Amsterdam, The Netherlands).

### 4.7. Liver Histology

Liver tissue was fixed in 4% formaldehyde for 24 h (Sigma-Aldrich) and deparaffinated with xylene, followed by rehydration with decreasing ethanol concentrations. Tissue sections (3–5 μm in thickness) were stained with periodic acid-Schiff (PAS). Lipidosis and extramedullary hematopoiesis (EMH) were analyzed by a certified mouse pathologist in a blinded manner (0, no abnormality; 1, minimal abnormality; 2, mild abnormality; 3, moderate abnormality; 4, severe abnormality; 5, very severe abnormality).

### 4.8. Intraperitoneal Glucose and Insulin Tolerance Tests

After fasting for 16 h over night, 2 mg of glucose/g (20% *w/v*; Braun, Hessen, Germany) or 0.75 IU/kg insulin (Lilly Humalog, Indianapolis, IN, USA) were intraperito-neally injected. Blood samples from the tip of the tail were analyzed after the indicated time points using a StatStrip Xpress glucose meter (Nova Biomedical).

### 4.9. ELISAs

Basal insulin levels were determined in plasma taken from mice starved for 4 h during the light cycle using an anti-insulin enzyme-linked immunosorbent assay (ELISA) following the manufacturer’s description (Mercodia, Uppsala, Sweden). Thyroid-stimulating hormone (TSH) plasma values were analyzed with an anti-TSH ELISA following the manufacturer’s description (Cloud-clone Corporation, Katy, TX, USA). Absorbance was determined with a microplate reader (Infinite 200 Pro, Tecan, Maennedorf, Switzerland).

### 4.10. Body Composition Analysis

An EchoMRI whole-body composition analyzer was used to analyze lean and fat mass non-invasively according to the manufacturer’s instructions (Echo Medical systems, Houston, TX, USA).

### 4.11. Indirect Calorimetry

Animals were single housed in acrylic glass airtight metabolic cages attached to an open calorimetry system (TSE Systems, Bad Homburg, Germany). Food and water were provided ad libitum. O_2_ consumption and CO_2_ production were measured during 3 days and the average was calculated. Energy expenditure was calculated according to the Weir equation and normalized to the corresponding lean mass + 0.2 times the fat mass, as determined by body composition analysis by EchoMRI [48,49].

### 4.12. Food/Water Intake, Urine/Feces Production

Mice were single housed in metabolic cages with grid surfaces (Techniplast, Schwerzenbach, Switzerland) and food, water, urine, feces and BW were monitored every 24 h for 72 h. Urine was collected under light mineral oil (Sigma-Aldrich) to avoid evaporation.

### 4.13. Statistical Analysis

Two-tailed Student’s *t*-test or Mann–Whitney test were applied for the analysis of two different data points as indicated. Comparison of more than two data points were carried out by two-way ANOVA followed by Bonferroni post-test.

## Data Availability

All main data of this study are included in the main part of the manuscript or supplementary materials. All underlying raw data have been made available on https://dataverse.harvard.edu/ via the accession code https://doi.org/10.7910/DVN/5OFBZR, accessed on 4 January 2022.

## Supplementary Materials

Supplementary Materials are available online at www.mdpi.com/article/10.3390/ijms23031536/s1.
