# Supplementary material for: The Deubiquitinase OTUB1 Is a Key Regulator of Energy Metabolism
Source: Int J Mol Sci. 2022 Jan 28;23(3):1536. doi: 10.3390/ijms23031536 (PMC8836018; doi:10.3390/ijms23031536)
Supplement: Supplementary file 1 [file ijms-23-01536-s001.zip › ijms-1565677-supplementary.pdf]

Supplementary Material

The deubiquitinase OTUB1 is a key regulator of energy metabolism

Amalia Ruiz-Serrano[1], Christina N. Boyle[2], Josep M. Monné Rodríguez[3], Julia Günter[1,4], Agnieszka E. Jucht[1], Svende Pfundstein[1], Andreas M. Bapst[1], Thomas A. Lutz[2], Roland H. Wenger[1,4,*], and Carsten C. Scholz[1,4,*]

[1]Institute of Physiology, University of Zurich, Zurich, Switzerland. [2]Institute of Veterinary Physiology, University of Zurich, Zurich, Switzerland. [3]Laboratory for Animal Model Pathology (LAMP), Institute of Veterinary Pathology, University of Zurich, Zurich, Switzerland. [4]National Centre of Competence in Research 'Kidney.CH', Switzerland.

Genotyping PCR primers

Otub1$^{fl/fl}$:

Ef_5272: 5'-TCCACCCCTTCATCCTGCTTTCT-3'

Er_5277: 5'-CAGACCAGAGCAGGATTAAGAAGCCTA-3'

UBC-Cre^{ERT2}:

UBCre 25285: 5'-GACGTCACCCGTTCTGTTG-3'

UBCre-oIMR7338: 5'-CTAGGCCACAGAATTGAAAGATCT-3'

UBCre-oIMR7339: 5'-GTAGGTGGAAATTCTAGCATC-3'

UBCre-oIMR9074: 5'-AGGCAAATTTTGGTGTACGG-3'

Otub1 iKO:

L3r_ 5274: 5'-TCTACCCATCCCAACACCAGCAAG-3'

Ef_5273: 5'-GAAGGACAAAGGGCGTGTCTCAGT-3'

loxP:

Primer 1307 forw: 5'-GGCAGAAGCACGCTTATCG-3'

Primer 1307 rev: 5'-GACAAGCGTTAGTAGGCACAT-3'

Supplementary Figures

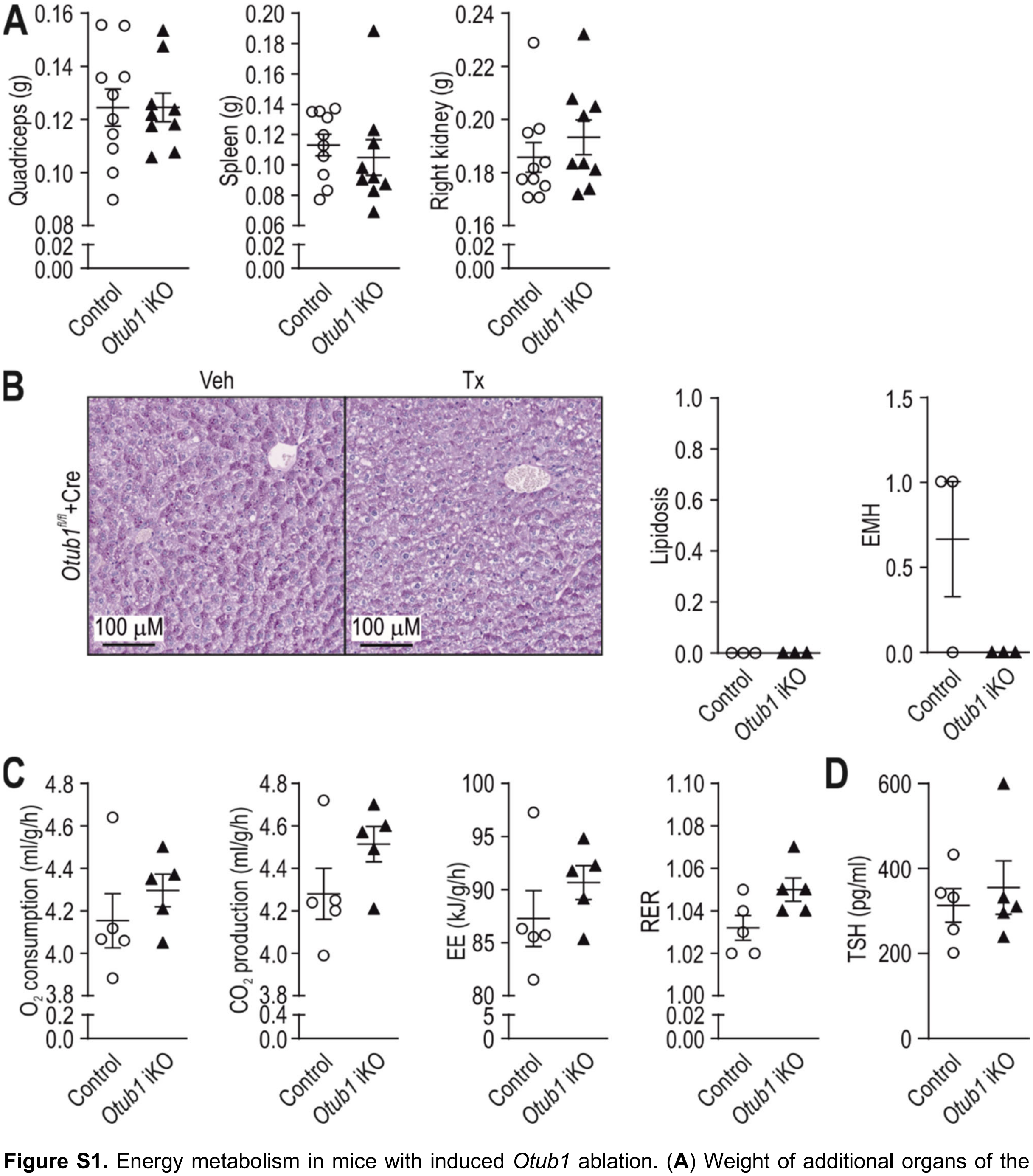

Figure S1. Energy metabolism in mice with induced *Otub1* ablation. (**A**) Weight of additional organs of the same mice shown in Figure 1C. (**B**) Representative periodic acid-Schiff (PAS) staining of transversal liver slices with scored lipidosis levels and extramedullary hematopoiesis (EMH) (n=3). (**C**) Averaged O_2 consumption, CO_2 production, energy expenditure (EE) and respiratory exchange ratio (RER) of 3 consecutive days during the night cycle (n=5). (**D**) Thyroid-stimulating hormone (TSH) plasma levels (n=5). All analyses were performed 5 months after *Otub1* deletion in 6.5 months old mice. Data are shown as mean ± SEM.

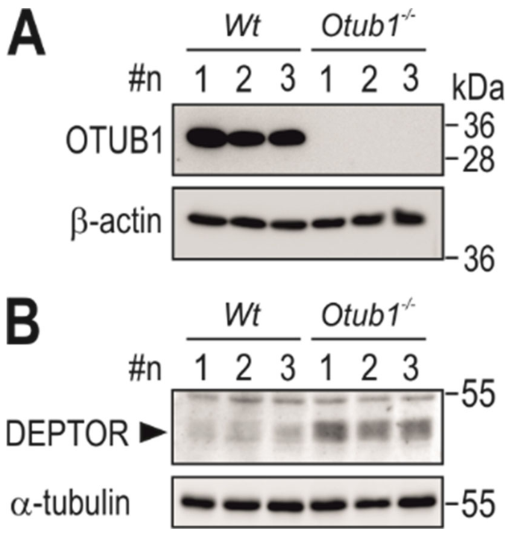

Figure S2. Generation of *Otub1*$^{-/-}$ mouse embryonic fibroblasts. (**A-B**) Immunblotting of the indicated proteins in wildtype (*Wt*) and *Otub1*$^{-/-}$ cell lysates; #n, n-number; exp., exposure. Full-length immunoblots are shown in Figure S9.

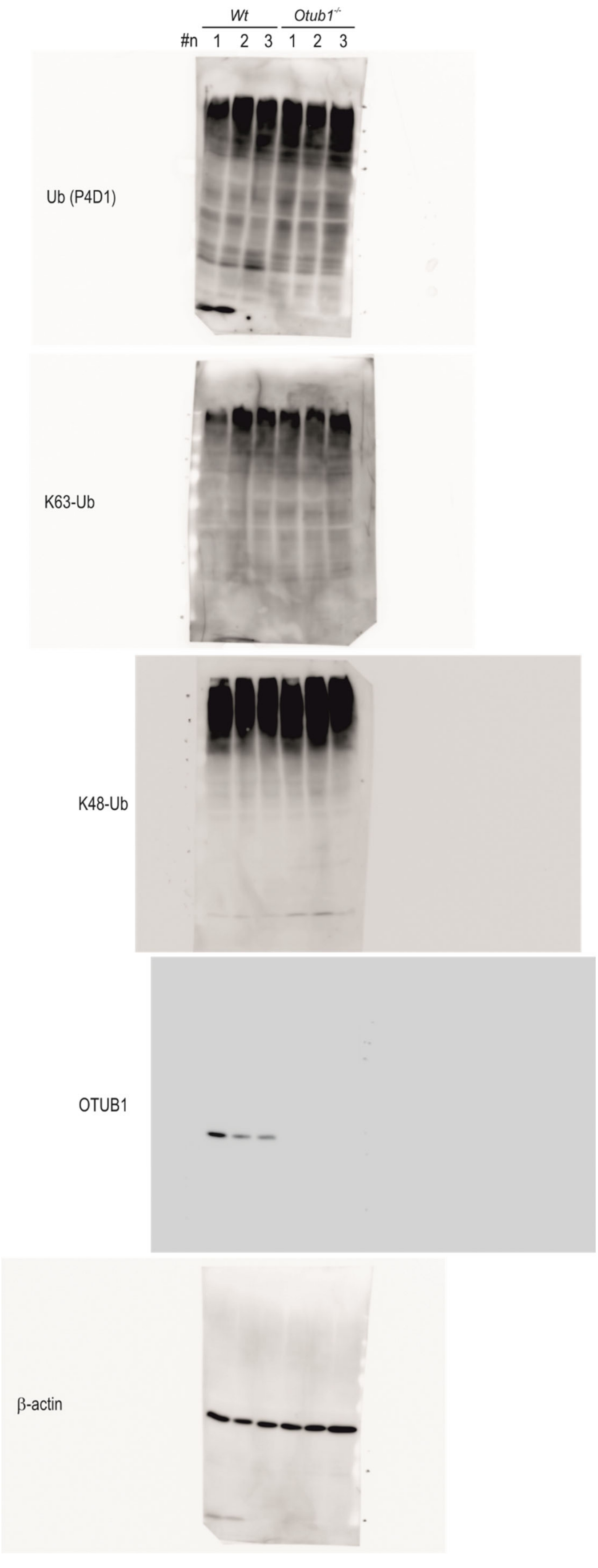

Figure S3. Full-length immunoblots of results shown in Figure 3A.

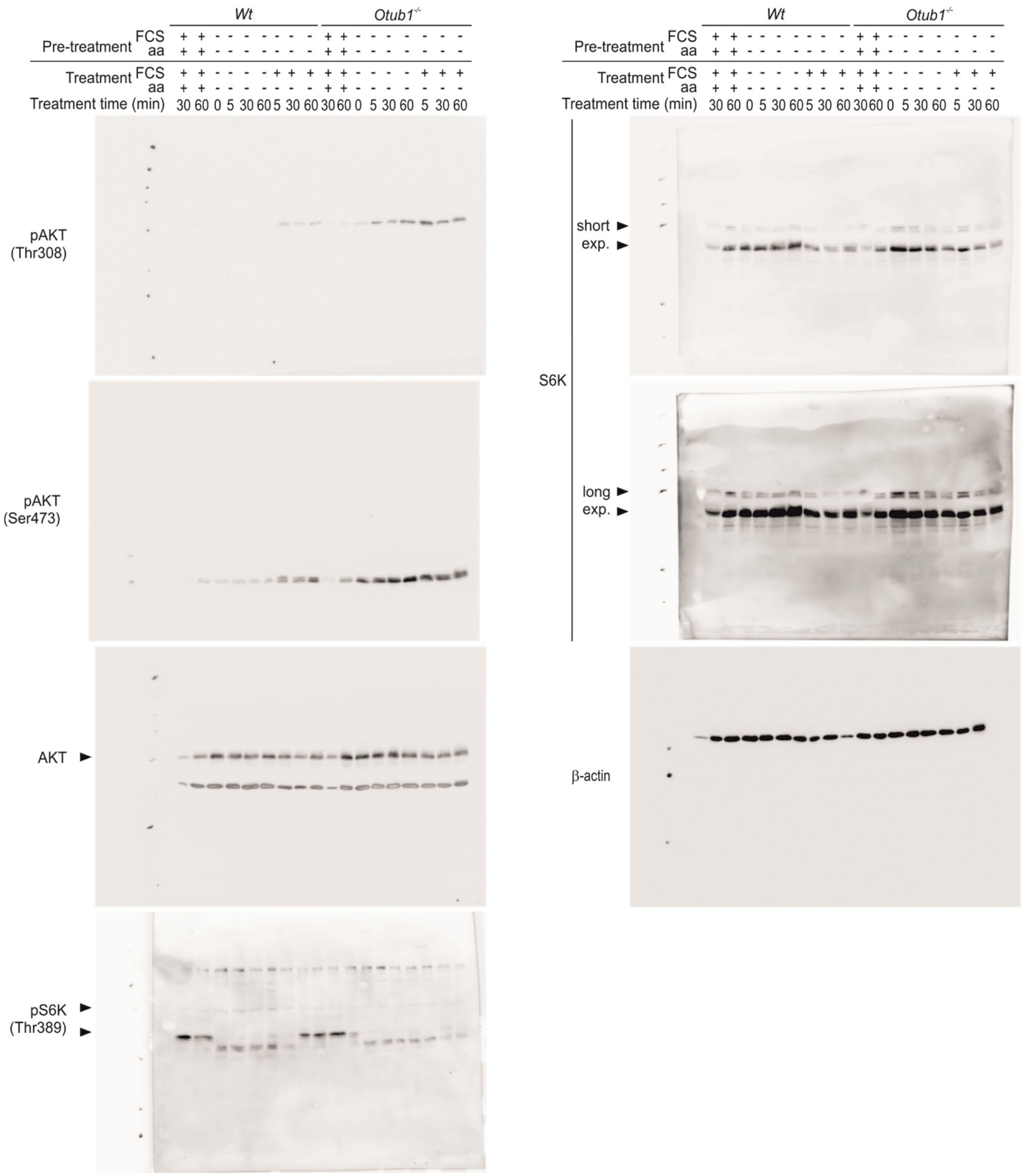

Figure S4. Full-length immunoblots of results shown in Figure 3B.

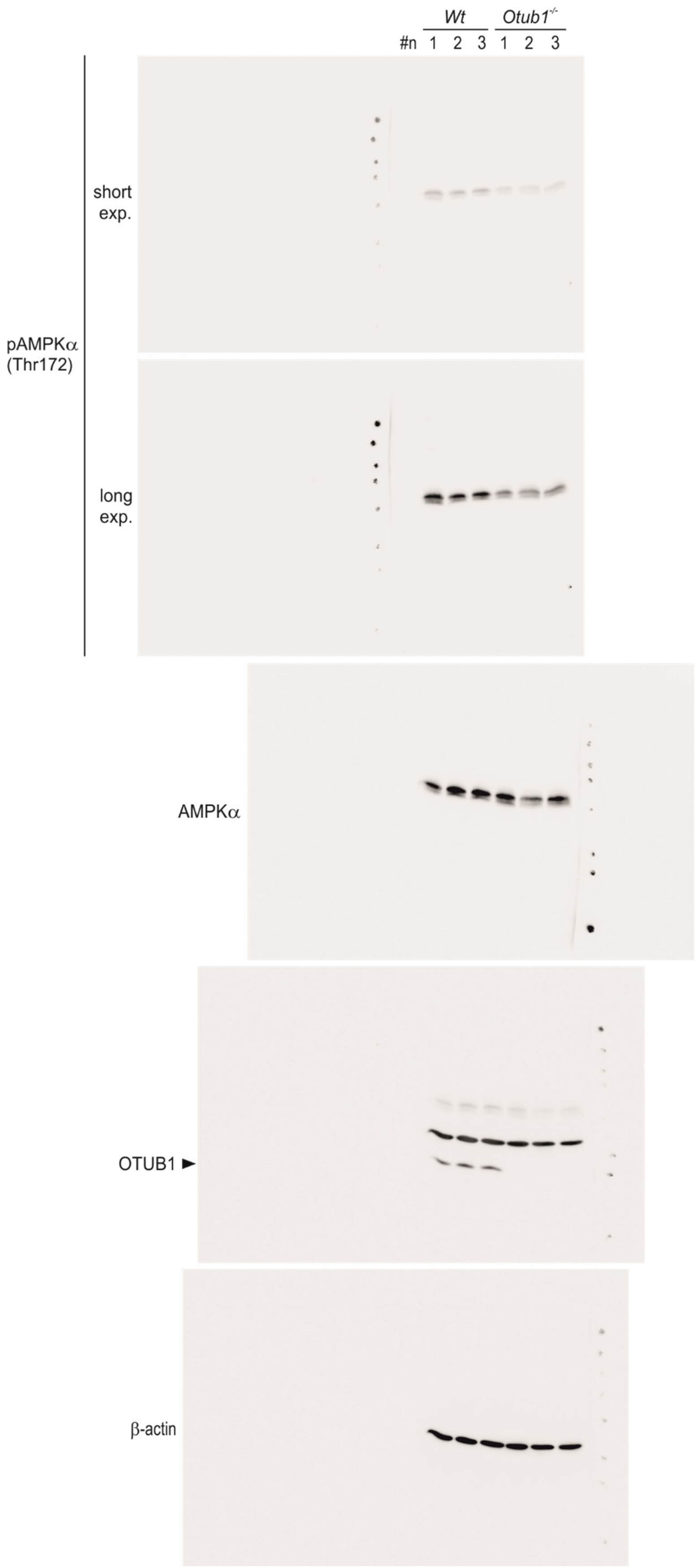

Figure S5. Full-length immunoblots of results shown in Figure 3F.

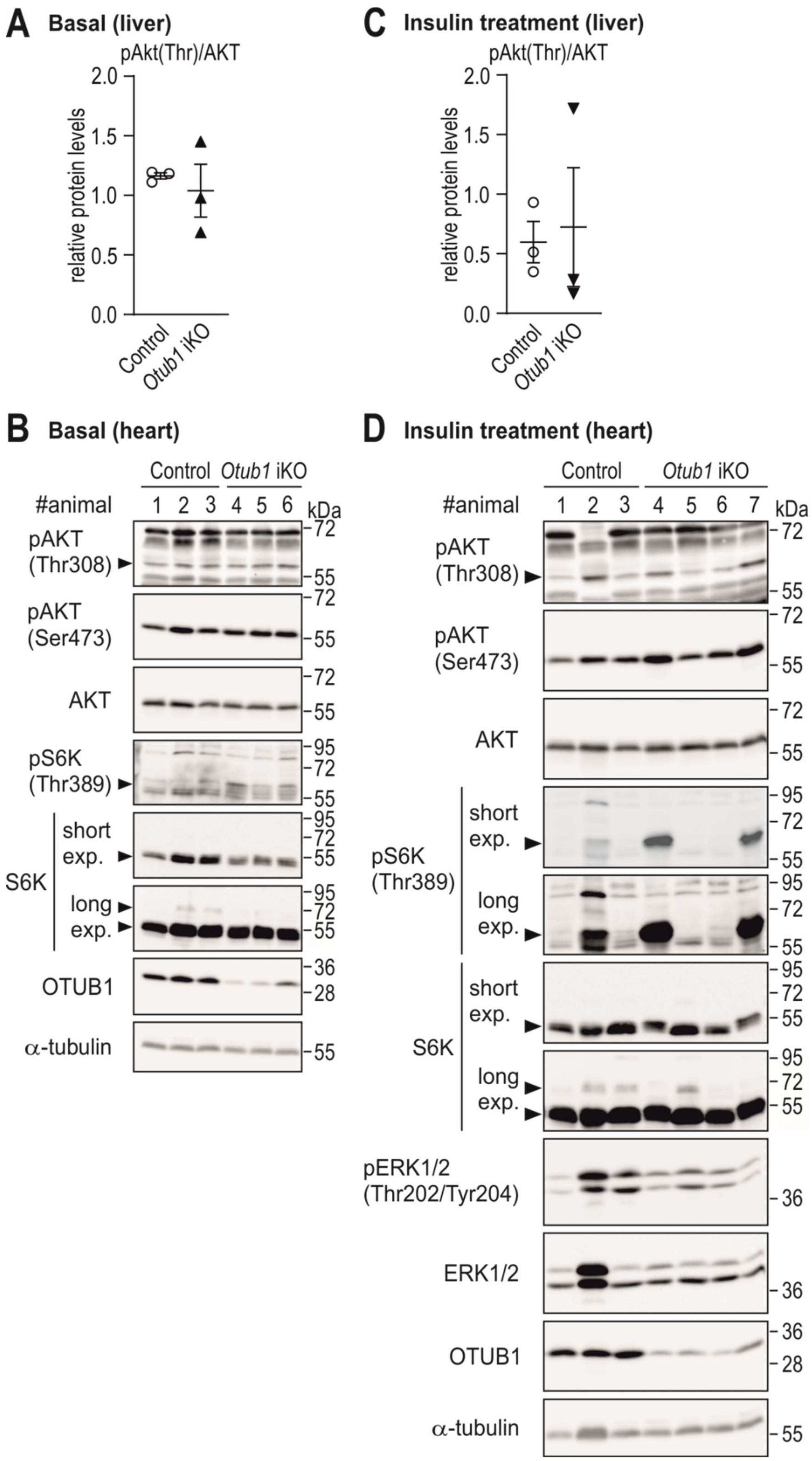

Figure S6. Protein levels in hearts of *Otub1* iKO mice. (**A**, **C**) Quantification of the indicated immunoblots shown in Figure 5 (normalized to loading control and the indicated protein). (**B**) Immunoblotting of the indicated proteins in heart lysates from control (with/without Cre and vehicle/tamoxifen treatment) and *Otub1* iKO mice (6 months after *Otub1* deletion; 8.5 months old; n = 3). (**D**) 10 days after *Otub1* KO (2 months old), mice were starved overnight (16 h) followed by insulin IP application (0.75 mU insulin/g) 15 min prior to tissue harvest (control, n = 3; *Otub1* iKO, n = 4). Heart lysates were analysed by immunoblotting. #animal, animal number. Full-length immunoblots are shown in Figures S10 and S11.

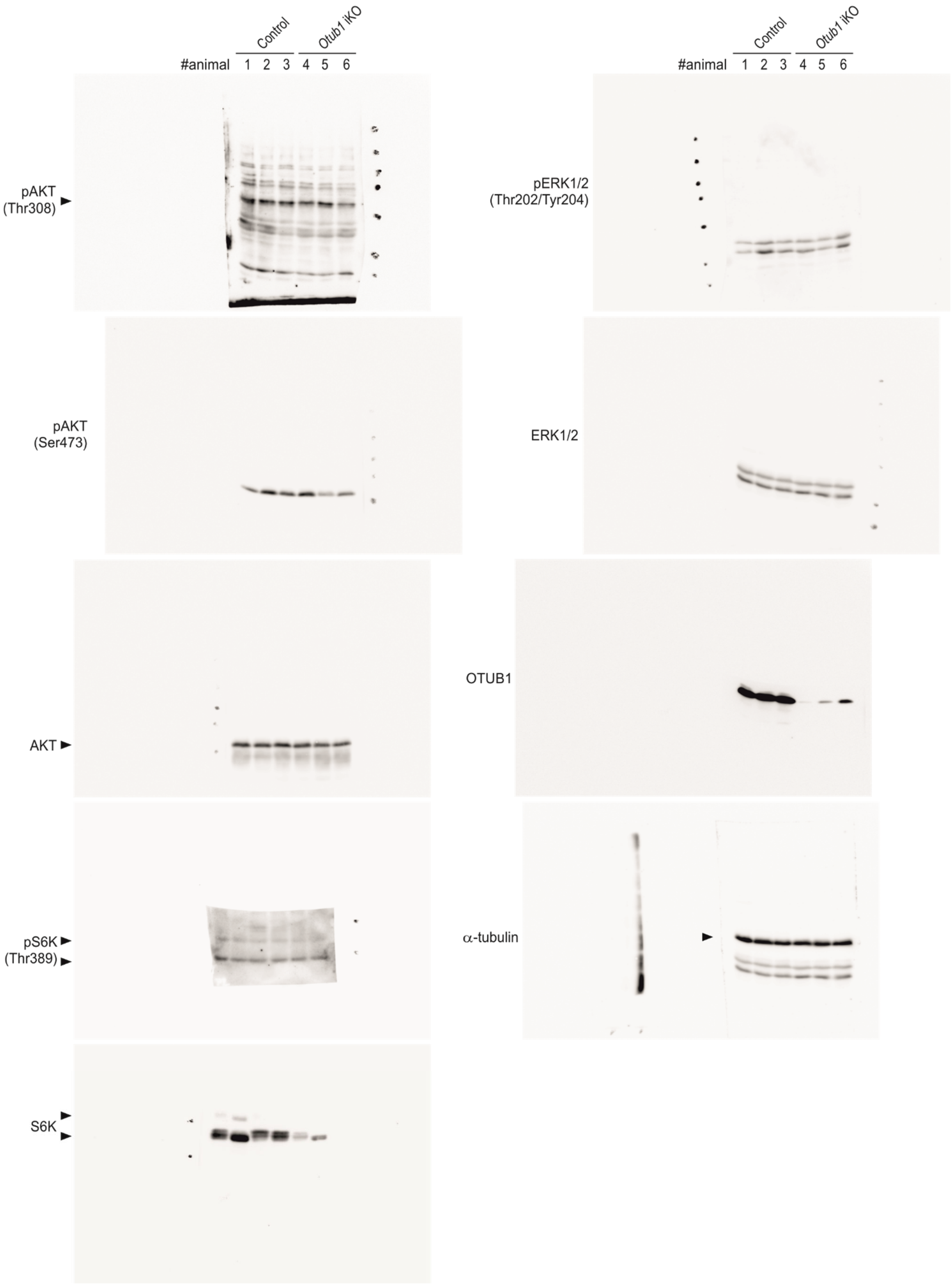

Figure S7. Full-length immunoblots of results shown in Figure 5A.

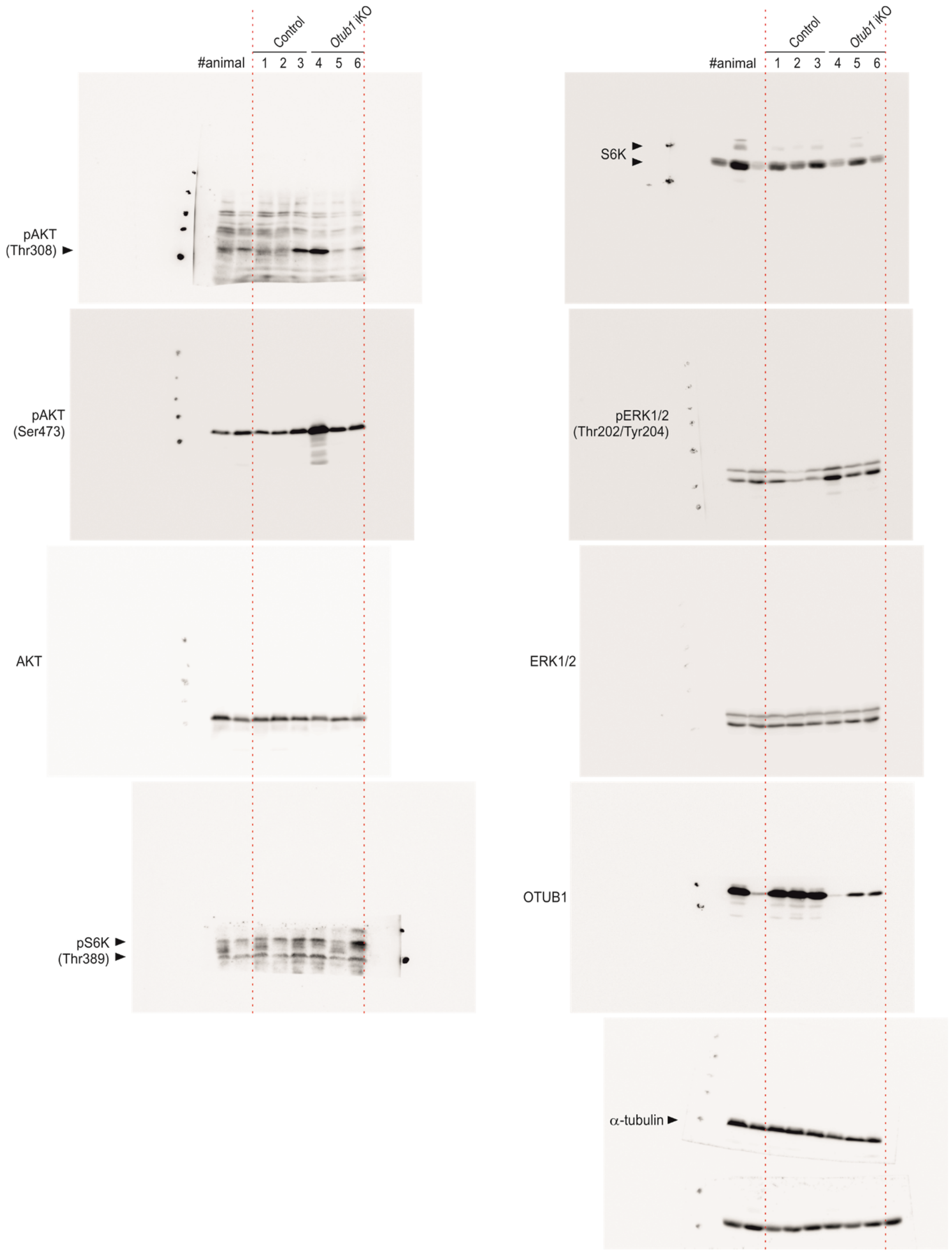

Figure S8. Full-length immunoblots of results shown in Figure 5B.

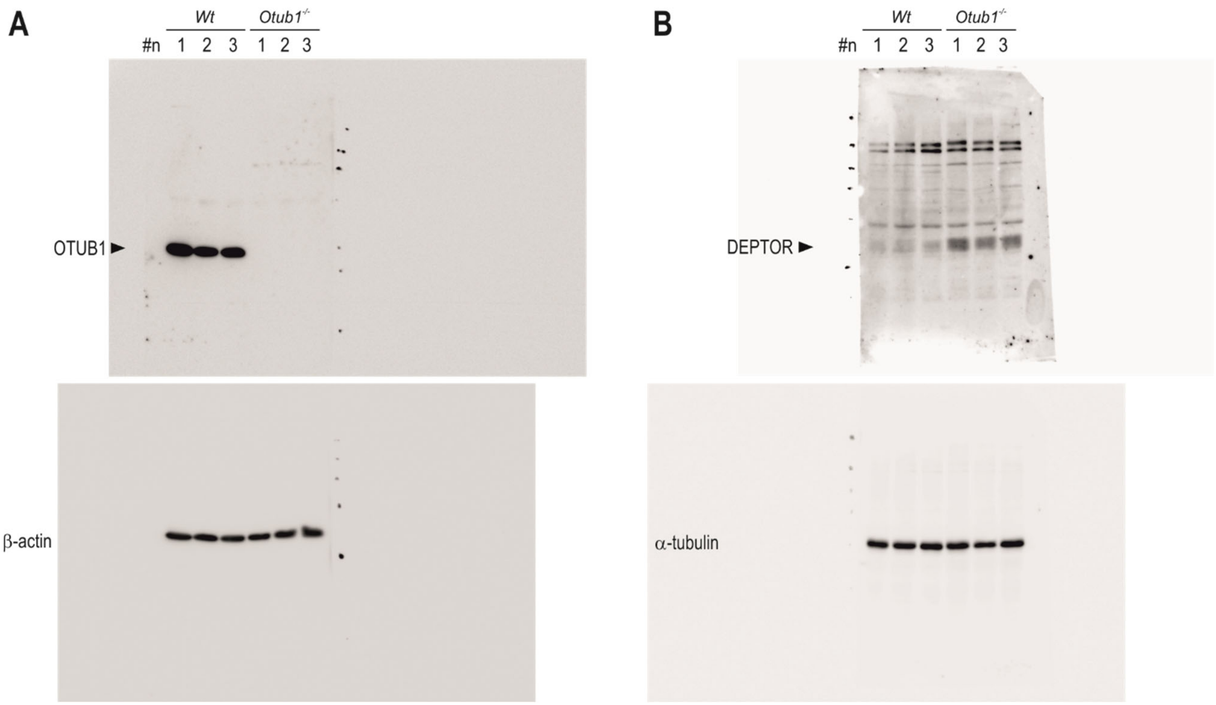

Figure S9. Full-length immunoblots of results shown in Figure S2.

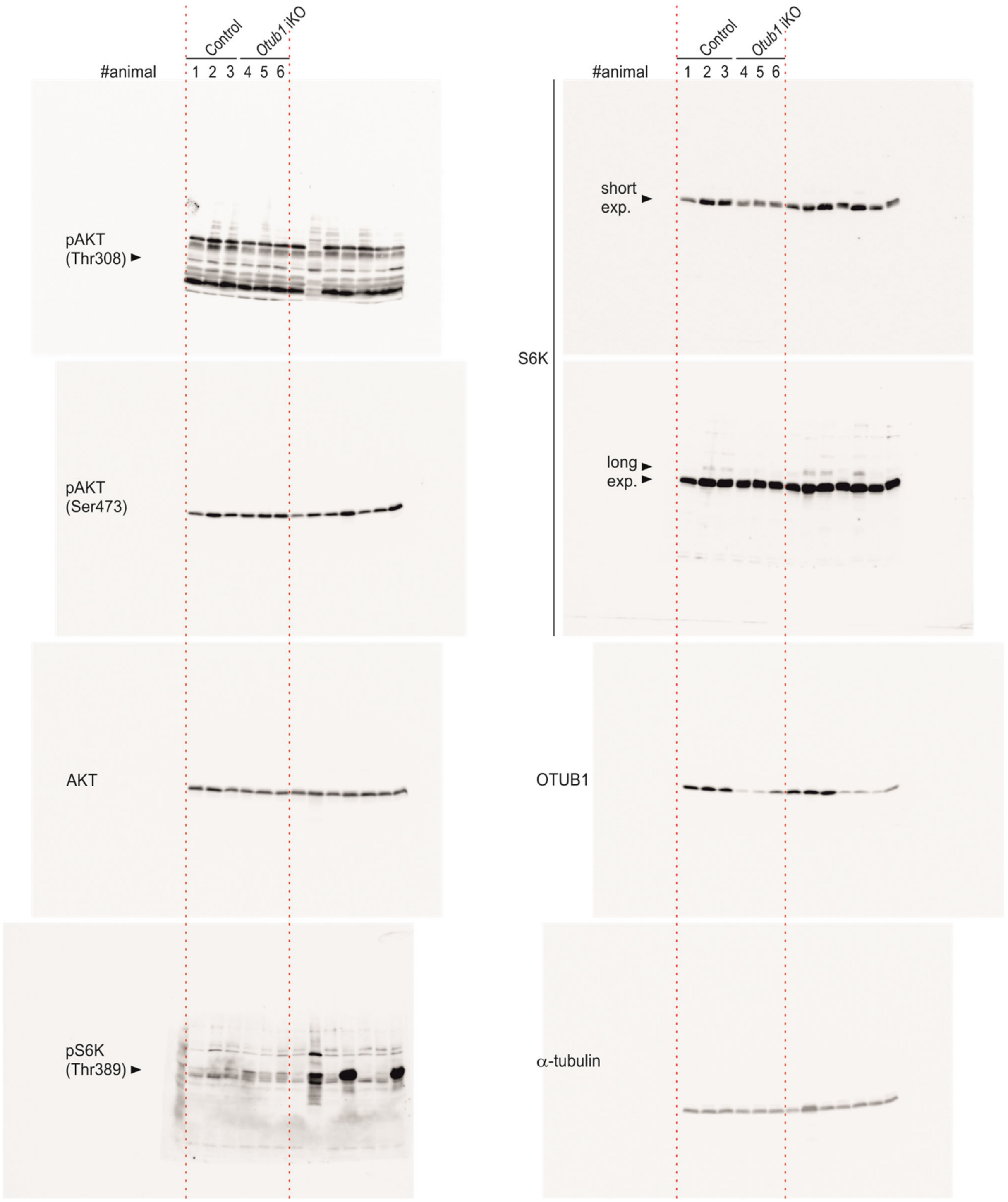

Figure S10. Full-length immunoblots of results shown in Figure S6A.

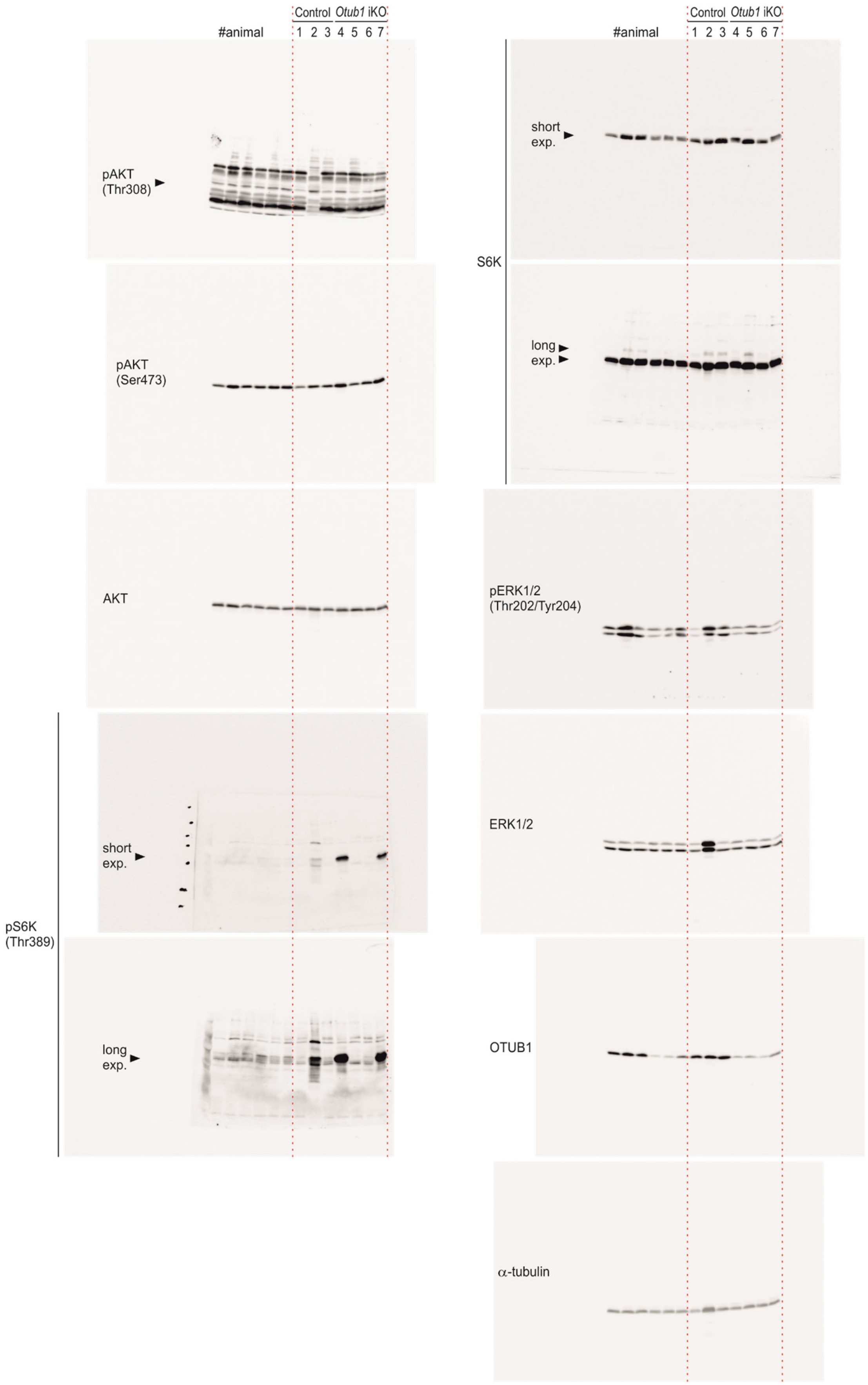

Figure S11. Full-length immunoblots of results shown in Figure S6B.
